# Human cardiomyocyte calcium handling and transverse tubules in mid‐stage of post‐myocardial‐infarction heart failure

**DOI:** 10.1002/ehf2.12271

**Published:** 2018-02-12

**Authors:** Morten Andre Høydal, Idar Kirkeby‐Garstad, Asbjørn Karevold, Rune Wiseth, Rune Haaverstad, Alexander Wahba, Tomas L. Stølen, Riccardo Contu, Gianluigi Condorelli, Øyvind Ellingsen, Godfrey L. Smith, Ole J. Kemi, Ulrik Wisløff

**Affiliations:** ^1^ Department of Circulation and Medical Imaging, Faculty of Medicine and Health Norwegian University of Science and Technology (NTNU) Trondheim Norway; ^2^ K.G. Jebsen Center of Exercise in Medicine Norwegian University of Science and Technology (NTNU) Trondheim Norway; ^3^ St. Olavs University Hospital Trondheim Norway; ^4^ Department of Cardiovascular Medicine, Humanitas Research Hospital CNR (National Research Council of Italy) Humanitas University Milan Italy; ^5^ Institute of Cardiovascular and Medical Sciences and School of Life Sciences, College of Medical, Veterinary, and Life Sciences, University of Glasgow Glasgow UK; ^6^ School of Human Movement and Nutrition Sciences University of Queensland Brisbane Australia

**Keywords:** Heart failure, Myocardial infarction, Calcium handling, Cardiomyocytes, Excitation contraction coupling, SERCA2a

## Abstract

**Aims:**

Cellular processes in the heart rely mainly on studies from experimental animal models or explanted hearts from patients with terminal end‐stage heart failure (HF). To address this limitation, we provide data on excitation contraction coupling, cardiomyocyte contraction and relaxation, and Ca^2+^ handling in post‐myocardial‐infarction (MI) patients at mid‐stage of HF.

**Methods and results:**

Nine MI patients and eight control patients without MI (non‐MI) were included. Biopsies were taken from the left ventricular myocardium and processed for further measurements with epifluorescence and confocal microscopy. Cardiomyocyte function was progressively impaired in MI cardiomyocytes compared with non‐MI cardiomyocytes when increasing electrical stimulation towards frequencies that simulate heart rates during physical activity (2 Hz); at 3 Hz, we observed almost total breakdown of function in MI. Concurrently, we observed impaired Ca^2+^ handling with more spontaneous Ca^2+^ release events, increased diastolic Ca^2+^, lower Ca^2+^ amplitude, and prolonged time to diastolic Ca^2+^ removal in MI (P < 0.01). Significantly reduced transverse‐tubule density (−35%, P < 0.01) and sarcoplasmic reticulum Ca^2+^ adenosine triphosphatase 2a (SERCA2a) function (−26%, P < 0.01) in MI cardiomyocytes may explain the findings. Reduced protein phosphorylation of phospholamban (PLB) serine‐16 and threonine‐17 in MI provides further mechanisms to the reduced function.

**Conclusions:**

Depressed cardiomyocyte contraction and relaxation were associated with impaired intracellular Ca^2+^ handling due to impaired SERCA2a activity caused by a combination of alteration in the PLB/SERCA2a ratio and chronic dephosphorylation of PLB as well as loss of transverse tubules, which disrupts normal intracellular Ca^2+^ homeostasis and handling. This is the first study that presents these mechanisms from viable and intact cardiomyocytes isolated from the left ventricle of human hearts at mid‐stage of post‐MI HF.

## Introduction

The prevalence of heart failure (HF) in Western countries is at present known to be over 23 million worldwide.^1^ In the industrialized part of the world, a common cause of HF is ischaemic heart disease, where a myocardial infarction (MI) often signals the onset of cardiac dysfunction that may progress to failure. HF is characterized by several abnormalities in the excitation–contraction coupling, such as reduced and slower systolic Ca^2+^ release from the sarcoplasmic reticulum (SR), elevated diastolic cytoplasmic Ca^2+^, and reduced diastolic Ca^2+^ removal, leading to reduced contractile function. Several mechanisms are responsible for this: disrupted cleft spacing between the L‐type Ca^2+^ channel and ryanodine receptors 2 (RyR2) by reduced density of transverse (T) tubules;^2^ increased RyR2 Ca^2+^ sensitivity leading to increased spontaneous Ca^2+^ release events from SR causing after‐depolarization and trigger arrhythmias;^3^ reduced SR Ca^2+^ adenosine triphosphatase 2a (SERCA2a) and increased Na^+^/Ca^2+^ exchanger activities.^4^ All these changes at the cellular level may explain the resulting breakdown of normal force–frequency relationships in HF, causing inadequate cardiac output responsiveness during physical effort.^5^ Attenuated SERCA2a response, with reduced SR Ca^2+^ uptake at increased stimulation frequencies, is especially important since this leads to a blunted frequency‐dependent acceleration of relaxation (FDAR), thereby impairing diastolic filling at high heart rates.^3^

However, all the presented findings are only predicted from experimental animal models and investigated in explanted human hearts at the terminal end stage of HF, a condition in which all interventions have failed to restore viable pump function. Despite research efforts on mechanisms of deteriorated cardiomyocyte contraction and relaxation and Ca^2+^ handling after MI, data from patients with post‐MI HF are limited, and especially, data from earlier pre‐terminal stages are almost absent. Given that coronary artery disease and MI are the most common causes of HF, data from the heart of patients with post‐MI HF at earlier stages of the disease are needed. When a targeted treatment focused on intracellular molecules and physiologic processes that directly cause dysfunction in HF is aimed for, it will be necessary to confirm mechanisms of failure as well as improvement in human cardiomyocytes at stages of HF where rescue may still be possible. Therefore, in order to determine cardiomyocyte contraction and relaxation, Ca^2+^ handling, and T‐tubule density in addition to key protein regulations involved in the progression towards heart failure, we analysed cardiomyocytes freshly isolated from the intact left ventricular myocardium of post‐MI patients at mid‐stages of HF.

## Methods

### Patient characteristics

Nine post‐MI patients with reduced left ventricle ejection fraction (EF < 35%) and eight patients without previous MI and normal EF (EF > 60%) (hereafter designated as control patients) scheduled for elective coronary artery bypass grafting (CABG) at the Department of Cardiothoracic Surgery, St. Olavs University Hospital, Trondheim, Norway, took part in the study. All patients included were on optimal treatment (*Table* 1). The study was approved by the Regional Committee for Medical and Health Research Ethics, Norway (study ID: TRIM 158‐04; clinical trial registration information: NCT00218985). All patients gave informed consent to participate in the study.

**Table 1:** Physical characteristics of the patients at hospitalization before coronary artery bypass grafting

	Post‐myocardial‐infarction heart failure patients (*n* = 9)	Control without previous myocardial infarction (*n* = 8)
Men/women	9/0	7/1
Age	69.2 ± 6.2	61.4 ± 10.0
Body mass index	27.6 ± 5.2	28.9 ± 3.0
Systolic blood pressure, rest (mmHg)	126.4 ± 14.7	142.5 ± 18.3
Diastolic blood pressure, rest (mmHg)	68.3 ± 11.5	79.4 ± 12.1
Work load (W)^a^	87.5 ± 17.7^b^	133.3 ± 43.8
Ejection fraction	30.1 ± 3.3^b^	72.4 ± 10.4
Number of previous myocardial infarctions		
One	6	None
Two	2	None
Three	1	None
New York Heart Association class of functional capacity^c^		
I	0	1
II	2	4
III	6	3
IV	1	0
Diabetes mellitus	3/9	1/8
Medications		
Beta‐blockers	7/9	8/8
Angiotensin‐converting enzyme inhibitors	7/9	2/8
Ca^2+^ channel blockers	1/9	3/8
Diuretics	6/9	1/8

a Work load during clinical evaluation of stress test electrocardiogram.

b Significantly different from patients without previous myocardial infarction (*P* < 0.01).

c The patients were clinically characterized according to the New York Heart Association classification of functional capacity and objective assessment of patients with diseases of the heart (defined both by classifications of angina pectoris symptoms and by symptoms of dyspnoea and left ventricle failure^55^). In the control patients without previous myocardial infarction, the New York Heart Association functional classification was only defined by their symptoms of angina as neither signs of dyspnoea nor left ventricular failure was present, whereas the patients with myocardial infarction also were limited by dyspnoea and symptoms of left ventricular failure.

### Biopsy sampling

All biopsies were sampled after sternotomy and pericardiotomy during CABG surgery. Biopsies were taken before aortic cross‐clamping from non‐fibrotic and viable left ventricle mid‐myocardium in the remote non‐infarcted area between the apex and base using a BioPince™ needle from Angiotech Pharmaceuticals, Inc (Vancouver, Canada). All perioperative procedures were performed according to standard routines of the department. Biopsies were put into an ice‐cold stabilizing physiological solution for further processing of cells and fibres within 20–30 min, whereas biopsies for biochemical analyses were snap‐frozen in liquid nitrogen within 30 s.

### Cardiomyocyte isolation

The biopsies were put into a Ca^2+^‐free HEPES‐based solution for free dissection of fibres by fine forceps under a microscope. Samples were then transferred to 95% O_2_ and 5% CO_2_ gassed Ca^2+^‐free Krebs–Henseleit solution with collagenase Type 2 (Worthington, Lakewood, NJ) and essential fatty‐acid‐free bovine serum albumin (Sigma) that was shaken for 20 min at 37°C. Next, the biopsies were filtered through a 150 μm masked nylon mesh and thereafter centrifuged at 60 *g* at 20°C for 30 s, whereafter the supernatant was removed and the pellet resuspended in a HEPES‐based solution with a stepwise increase in Ca^2+^ concentration (0.1–1.8 mmol/L) with centrifugation between each step.

### Cardiomyocyte shortening and Ca^2+^ cycling

Fura‐2/AM‐loaded (2 μmol/L, Molecular Probes, Eugene, OR) cardiomyocytes were stimulated by bipolar electrical pulses with increasing frequency (1–3 Hz) on an inverted epifluorescence microscope (Nikon TE‐2000E, Tokyo, Japan), whereupon cell shortening was recorded by video‐based myocyte sarcomere spacing (SarcLen™, IonOptix Corporation, MA). Intracellular Ca^2+^ concentration was measured by counting 510 nm emission with a photomultiplier tube (PMTACQ, IonOptix, Milton, MA) after exciting with alternating 340 and 380 nm wavelengths (F^340/380^ ratio) (OptoScan, Cairn Research Ltd, Kent, UK). During the stimulation protocol, cells were continuously perfused with a normal physiological HEPES‐based solution (1.8 mmol/L Ca^2+^) in room temperature of 22 ± 0.5°C.

### Transverse‐tubule density recordings

Isolated quiescent cardiomyocytes loaded with the membrane‐specific Di‐8‐ANEPPS dye (10 μmol/L for 20 min, Molecular Probes) were confocal imaged to study T‐tubules throughout the cell. The relative density of T‐tubules normalized to cell size was obtained from five images per cell, captured from the middle of each cell and analysed using a custom‐made application in IDL 6.0 (ITT Visual, Boulder, CO, USA), by counting pixels stained with the dye relative to the total number of pixels after removing pixels associated with the non‐T‐tubular sarcolemma.

### Sarcoplasmic reticulum Ca2+ adenosine triphosphatase 2a Ca^2+^ uptake measurements

Biopsy samples were excised and permeabilized by saponin (50 μg/mL, for 30 min, at 4°C), before being transferred to an adenosine triphosphate solution containing (mmol/L) 0.05 egtazic acid, 5 adenosine triphosphate, 10 CrP, 25 HEPES, 100 KCl, and 5.5 MgCl. We added Fura‐2 (10 μM; Molecular Probes) to probe Ca^2+^, oxalate (10 mM) to stabilize intra‐SR Ca^2+^, and ruthenium red (3 μM) to inhibit SR Ca^2+^ efflux and mitochondrial partitioning. The experiment was initiated by stirring tissue samples in a 150 μL cuvette while monitoring extra‐SR Ca^2+^ using the optical system described above and adding 50 μM Ca^2+^. Ca^2+^ loading induced an immediate increase in Ca^2+^ concentration, followed by a decline that was attributed to SERCA2a Ca^2+^ uptake. The ratio signal was converted to total Ca^2+^ concentration by measuring *R*_min_ and *R*_max_ in each tissue sample, and the rate of SR Ca^2+^ uptake was subsequently plotted against the corresponding free Ca^2+^. A logistic curve was fitted to estimate maximal Ca^2+^ uptake rate (*V*_max_).

### Protein expression and phosphorylation

Biopsies were homogenized with radioimmunoprecipitation assay buffer [10 mM Tris–HCl, pH 7.5, 150 mM KCl, 0.1% sodium dodecyl sulfate, 1% NP‐40, 5 mM ethylenediaminetetraacetic acid, protease and phosphatase inhibitor cocktails (Roche, Indianapolis, IN)] and centrifuged for 10 min at 20 000 *g* at 4°C, whereupon the supernatant was removed. The lysates were then loaded onto 8% SDS‐PAGE for detection of phospho‐Thr‐286‐CaMKII (Affinity Bioreagents, Golden, CO) and SERCA2a (ABR, Rockford, IL) and on 12% SDS‐PAGE for total phospholamban (PLB), phospho‐Thr‐17‐PLB, and phospho‐Ser16‐PLB (Badrilla, Leeds, UK). α‐Actin (Chemicon‐Upstate, Charlottesville, VA) was used to normalize protein levels. Proteins were transferred onto nitrocellulose membranes (Bio‐Rad, Hercules, CA), and membranes were blocked with TBS‐T/milk for 1 h at room temperature followed by overnight incubation with the mentioned antibodies. For protein detection, goat antirabbit or antimouse horseradish‐peroxidase‐conjugated secondary antibodies (Amersham GE, Freiburg, Germany) and enhanced chemiluminescence (Thermo Fisher Scientific Inc, Rockford, IL) were used. Densitometric analysis was performed using ImageJ software (NIH, Bethesda, MD).

### Statistical analysis

Data are shown as mean ± standard deviation unless otherwise stated. The Mann–Whitney U test was used to identify statistical differences between the groups. *P* < 0.05 was considered statistically significant.

## Results

**Figure 1:**
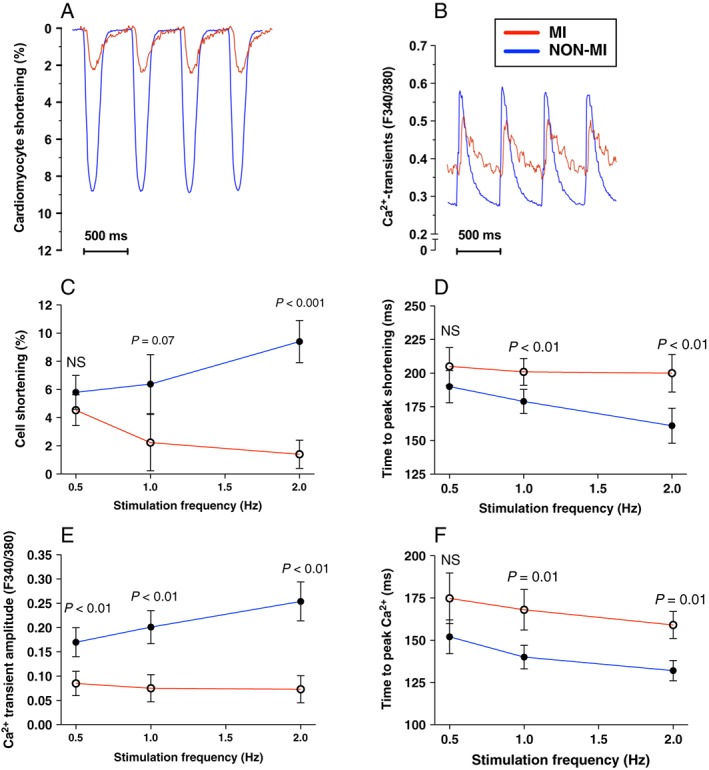
Cardiomyocyte function and Ca^2+^ handling. Example recordings of cardiomyocyte contraction–relaxation (A) and Ca^2+^ transients (B) at 2 Hz stimulation in post‐myocardial‐infarction heart failure patients (MI) (*N* = 9, *n* cells per patient: 6–10) (red lines) vs. non‐myocardial‐infarction patients (NON‐MI) (*N* = 8, *n* cells per patient: 6–10) (blue lines), reported by edge detection microscopy and Fura‐2/AM ratio (F340/380), respectively. (C) Cardiomyocyte fractional shortening. (D) Time to peak shortening. (E) Ca^2+^ transient amplitude. (F) Rates of Ca^2+^ release (time to peak Ca^2^ transient amplitude).

Human cardiomyocyte fractional shortening was similar between patients with non‐MI and MI at 0.5 Hz stimulation frequency (i.e. 30 b.p.m.), but in contrast to cardiomyocytes from non‐MI, we found a negative shortening–frequency relationship in MI cardiomyocytes: at a 2 Hz stimulation rate, cardiomyocyte shortening was significantly depressed (*P* < 0.001, *Figure* *1*). Rates of contraction were also reduced, as time to peak shortening was significantly longer at 1–2 Hz (12% and 26%, respectively, *P* < 0.01, *Figure* *1*). Ca^2+^ transient amplitude was significantly impaired at 0.5–2 Hz stimulation in MI patients, with as much as a ~45% reduction at 2 Hz (*P* < 0.01, *Figure* *1*). The rate of Ca^2+^ release (time to peak Ca^2+^ transient amplitude) was markedly slower, with a difference of 15% (not significant) at 0.5 Hz, increasing to 20% and 21% at 1 and 2 Hz, respectively (*P* < 0.01, *Figure* *1*).

**Figure 2:**
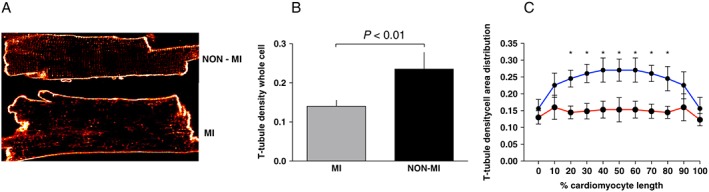
Transverse (T)‐tubule structure. T‐tubule density in post‐myocardial‐infarction heart failure patients (MI; *N* = 9, *n* cells per patient: 6–10) vs. non‐MI patients (NON‐MI; *N* = 8, *n* cells per patient: 6–10). (A) Example confocal images of T‐tubules in a di‐8‐ANEPPS‐stained cardiomyocyte from an MI patient. (B) Display of a significantly reduced T‐tubule density in MI vs. NON‐MI and (C) T‐tubule densities along relative cell length. The largest reduction in T‐tubule density of MI patients (red) compared with NON‐MI (blue) was found in the mid‐regions of the cardiomyocyte. Data are presented as mean ± standard deviation. *P < 0.01.

Because rapid and controlled activation of the Ca^2+^ transient amplitude is at least partly determined by the cleft area between the SR and plasma membranes, we studied T‐tubule density as a measure of this property. Corresponding to the reduced Ca^2+^ transient amplitude, we report a ~35% reduction in the T‐tubule density in MI cells (*P* < 0.01, *Figure* *2* *A*,*B*), where the most marked reduction was found in the mid‐regions of the cardiomyocyte (*Figure* *2* *C*).

**Figure 3:**
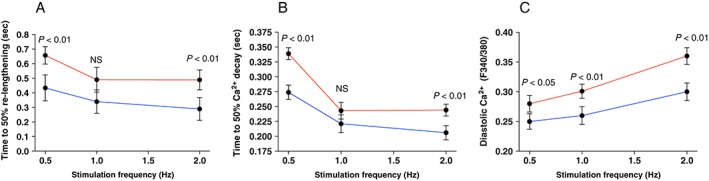
Cardiomyocyte diastolic function and Ca^2+^ handling. Cardiomyocyte diastolic function and Ca^2+^ handling properties at 0.5–2 Hz stimulation in patients with post‐myocardial‐infarction heart failure (*N* = 9, *n* cells per patient: 6–10) (red line) vs. non‐myocardial‐infarction patients (*N* = 8, n cells per patient: 6–10) (blue lines). (A) Frequency‐dependent acceleration of relaxation assessed by time to 50% diastolic re‐lengthening of the cardiomyocyte. (B) Frequency‐dependent acceleration of Ca^2+^ decay measured as time to 50% decay of the Ca^2+^ transient. (C) Diastolic cytoplasmic Ca^2+^ levels. Ca^2+^ transient tracings are reported with Fura‐2/AM ratio (F340/380).

The time from peak cardiomyocyte contraction to re‐lengthening provides a measure of diastolic cardiomyocyte relaxation. At 2 Hz stimulation, time to 50% re‐lengthening was 41% longer in MI cardiomyocytes, indicating severe diastolic dysfunction (*P* < 0.01, *Figure* *3* *A*).

Under normal conditions, negative FDAR with increasing heart rates accommodates adequate ventricular filling with shorter diastolic periods. We found negative FDAR in both non‐MI and MI cardiomyocytes at slow stimulation rates (0.5–1 Hz, i.e. from 30 to 60 b.p.m.). When stimulation frequency is increased further (1–2 Hz, i.e. from 60 to 120 b.p.m.), a marked shift occurred; whereas non‐MI cardiomyocytes further reduced time to 50% re‐lengthening, the MI cardiomyocytes did not (*Figure* *3* *A*), suggesting impaired adaptability at higher heart rates in these patients.

Cardiomyocyte relaxation during diastole is controlled by the removal of cytoplasmic Ca^2+^. Time to 50% Ca^2+^ decay was 33% (*P* < 0.01), 30% (not significant), and 40% (*P* < 0.01) longer at 0.5, 1, and 2 Hz, respectively, in MI cardiomyocytes (*P* < 0.01, *Figure* *3* *B*). Moreover, the effect of increasing stimulation frequencies on frequency‐dependent acceleration of Ca^2+^ decay was similar to those observed for FDAR, suggesting that observed differences between groups are due to abnormal Ca^2+^ handling, which was further evidenced by significantly higher diastolic Ca^2+^ levels in MI cardiomyocytes (*Figure* *3* *C*).

**Figure 4:**
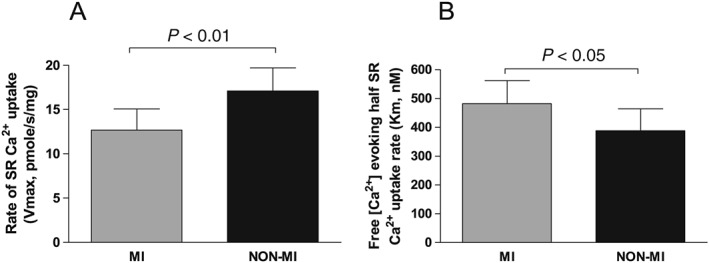
Sarcoplasmic reticulum (SR) Ca^2+^ adenosine triphosphatase 2a (SERCA2a) function. SERCA2a function assessed as SR Ca^2+^ uptake measurements in separate biopsies of the left ventricle myocardium in post‐myocardial‐infarction heart failure patients (MI) (*N* = 9) vs. non‐myocardial‐infarction patients (NON‐MI) (*N* = 8). (A) Maximal rate of SR Ca^2+^ uptake was lower in MI. (B) SERCA‐2a sensitivity to cytosolic Ca^2+^ (Km of free Ca^2+^ concentration evoking half SR Ca^2+^ uptake rate) was significantly lower in MI; higher Ca^2+^ levels are needed to activate SERCA2a.

In human cardiomyocytes, the main contributor to Ca^2+^ transient decay during diastole is SERCA2a.^6^ We measured SERCA2a pump function directly in separate tissue samples and found that the maximal rate of Ca^2+^ removal via SERCA2a was 26% lower in MI samples (*P* < 0.01, *Figure* *4* *A*), while Ca^2+^ sensitivity of SERCA2a was 24% reduced (*P* < 0.05 *Figure* *4* *B*). Cardiomyocyte cytosolic Ca^2+^ concentrations during diastole were significantly higher in MI hearts; 12% (*P* < 0.05), 16% (*P* < 0.01), and 20% (*P* < 0.05) at 0.5, 1, and 2 Hz, respectively (*Figure* *3* *C*).

**Figure 5:**
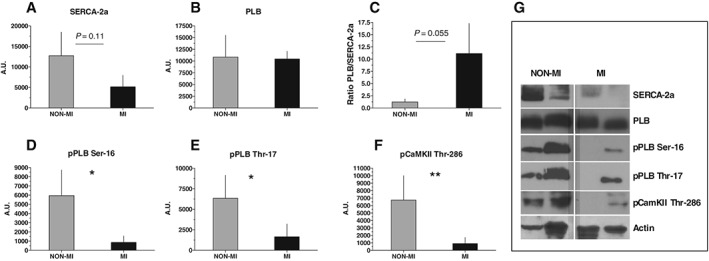
Protein expression. Western blot data from left ventricle biopsies in post‐myocardial‐infarction heart failure patients (MI) (*N* = 6) vs. non‐myocardial‐infarction patients (NON‐MI) (*N* = 5). (A) Sarcoplasmic reticulum Ca^2+^ adenosine triphosphatase 2a (SERCA2a) only showed a tendency of down‐regulation (*P* = 0.11) in MI patients. (B) Phospholamban (PLB) was not changed, but (C) the ratio PLB/SERCA2a revealed a tendency (*P* = 0.055) of increased levels in MI compared with NON‐MI. (D) Both the phosphorylation site of PKA at PLB serine‐16 (pPLB Ser‐16) and (E) the phosphorylation site of CaMKII at PLB theorine‐17 (pPLB Thr‐17) was significantly lower activated in MI patients. (F) Phosphorylated CaMKII at the auto‐activating site threonine‐286 (pCaMKII Thr‐286) was lower in MI. (G) Representative images of Western blot for each group. Data are presented as mean ± standard error of the mean. *P < 0.05, **P < 0.01.

Mechanisms were explored by measuring protein expression of SERCA2a and PLB, its innate inhibitor. Total SERCA2a protein levels and PLB/SERCA2a ratio revealed a tendency of lower levels in MI vs. non‐MI (*P* = 0.11 and 0.055, respectively) (*Figure* *5*). Notably, PLB phosphorylation was significantly reduced at both serine‐16 and threonine‐17 (*P* < 0.05, *Figure* *5*). This is important, as phosphorylation of PLB at either site leads to higher activity of SERCA2a. To further determine the upstream signalling of reduced PLB phosphorylation of PLB at Thr‐17, we found that the activity of CaMKII (measured by phosphorylated CaMKII at the Thr‐286 site) was significantly reduced in MI (*P* < 0.01, *Figure* *5*). In summary, these findings suggest a mechanism that could account for reduced cardiomyocyte Ca^2+^ transient decay rate and longer time to re‐lengthening.

**Figure 6:**
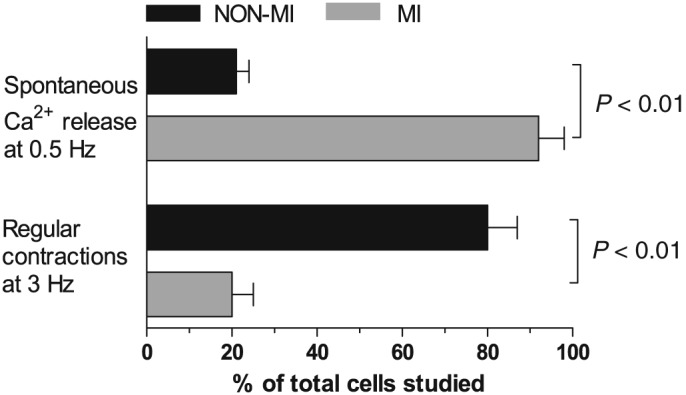
Spontaneous Ca^2+^ release and irregular cardiomyocyte activation. Percentage of cells with spontaneous Ca^2+^ release events between regular electrical stimulated twitch Ca^2+^ releases (upper graphs) was higher in post–myocardial‐infarction heart failure patients (MI) (*N* = 9, *n* cells per patient: 6–10) compared with non‐MI patients (NON‐MI) (*N* = 8, *n* cells per patient: 6–10). Lower graphs display that the cardiomyocytes from MI had impaired ability to follow electrical stimulation when increasing the frequency from 2 to 3 Hz.

Finally, MI cardiomyocytes had significantly more spontaneous Ca^2+^ releases between regular electrical stimulated contractions; as much as 90% of the total number of cells had spontaneous Ca^2+^ release events between regular twitch stimulations at 0.5 Hz, in contrast to only 20% in non‐MI cardiomyocytes (*P* < 0.01, *Figure* *6*). This observation implies that MI cardiomyocytes may be more prone to Ca^2+^‐mediated triggering of ventricular arrhythmias, which is further supported by the observation that MI cardiomyocytes show lower response to electrical stimulation from 2 to 3 Hz (corresponding to 180 b.p.m.); only 20% of MI cardiomyocytes followed stimulation at 3 Hz, in contrast to 80% in non‐MI (*P* < 0.01, *Figure* *6*).

## Discussion

The abnormalities in systolic and diastolic Ca^2+^ handling by alteration in several Ca^2+^ transporters and regulatory proteins are key mechanisms of contractile dysfunction in HF and have been extensively studied during the last decays (reviewed in several papers^7^, ^8^, ^9^, ^10^, ^11^). However, these studies and therefore our knowledge of cellular processes in the heart have relied mainly on experiments performed in animal models or explanted hearts from patients with terminal end‐stage HF in which heart transplantation is necessary or death is inevitable. This is an obvious limitation in existing knowledge, since data from earlier stages of the disease are more relevant for optimizing treatment. To address this limitation, we examined freshly isolated cardiomyocytes in biopsies from the remote non‐infarcted area of the left ventricle mid‐myocardium in hearts from post‐MI HF patients scheduled for CABG at the mid‐phase stage of HF. The procedure of biopsy sampling during CABG opens a window for detailed and quantitative assessment of cellular physiology of the human myocardium before it enters end‐stage HF, in a manner not previously available. The present data confirm that contractile dysfunction in post‐MI patients at mid‐stage of HF is at least partly caused by abnormalities in systolic and diastolic Ca^2+^ handling, by a loss of T‐tubules and hence the tight control of the structure of the cleft area between the plasma membrane and the SR, and by differential regulation in several Ca^2+^ transporters and regulatory proteins.

### Cardiomyocyte dysfunction

In healthy hearts, the increased peripheral demand for oxygenated blood is met by both increasing cardiac frequency and myocardial contractility, which is in contrast to failing hearts that cannot adapt to ventricular function normally with consequences of dyspnoea and fatigue.^12^ We show that the underpinnings to reduced cardiac function are present at the cellular level already at early stages of HF in post‐MI patients. Aggravation of cardiomyocyte contractility during increasing stimulation frequencies suggests that MI patients with reduced EF have somewhat preserved contractile capacity at rest, but a reduced ability to increase contractile capacity at higher heart rates such as during physical activity.

### Impaired cytosolic and sarcoplasmic reticulum Ca^2+^ handling

The corresponding changes of reduced contractility and intracellular Ca^2+^ handling in MI cardiomyocytes, and the link between these features,^10^ suggest that the observed differences in Ca^2+^ handling could subsequently explain reduced contractility. Reduced fractional shortening, rate of diastolic re‐lengthening, and FDAR in MI cardiomyocytes paralleled reduced Ca^2+^ transient amplitude, rate of Ca^2+^ transient decay, and frequency‐dependent acceleration of Ca^2+^ transient decay, respectively, which led us to perform several follow‐up experiments to support a mechanistic understanding of the phenotype. First, direct measurements of SR Ca^2+^ uptake in permeabilized myocardial fibres revealed reduced SERCA2a function in MI, which explains the reduced rates of Ca^2+^ transient decay. The observed reduction in SR Ca^2+^ uptake by SERCA2a may partly contribute to the observed increase in diastolic intracellular Ca^2+^ concentration in MI cardiomyocytes. Elevated diastolic Ca^2+^ may cause contractile dysfunction by preventing full relaxation of the myofilaments,^13^ as well as electrical disturbances, since it increases Ca^2+^ extrusion by the Na^+^/Ca^2+^ exchanger, which may increase the incidence of delayed after‐depolarizations and hence arrhythmic events.^3^ The increased frequency of spontaneous Ca^2+^ release events also observed in the present study would further accentuate arrhythmic events. Thus, impaired diastolic Ca^2+^ handling probably translates into both contractile and electrical abnormalities with detrimental clinical consequences such as reduced inotropy and higher susceptibility to ventricular arrhythmias in failing hearts. Furthermore, a lack of response to higher stimulation frequencies (3 Hz) substantiates deterioration of contractile and electrical properties in MI.

Despite the direct recordings of impaired SERCA2a function, we did, however, only observe a tendency towards reduced SERCA2a protein expression in the left ventricle from MI patients. Limited data exist on the regulation of SERCA2a from human sources, and all data are from explanted hearts at terminal end‐stage HF, that is, a substantially different cohort than the current patients. Notwithstanding this, controversies exist regarding regulation of SERCA2a proteins, where several reports show no change of SERCA2a protein in HF,^14^, ^15^, ^16^, ^17^, ^18^, ^19^, ^20^ and others show reduced protein levels of SERCA2a.^21^, ^22^, ^23^ Despite the controversies on the extent of SERCA2a protein regulation in human HF, a clinical trial with the aim of increasing the SERCA2a protein levels in patients with HF was designed using recombinant adeno‐associated virus Serotype 1/SERCA2a;^24^ the initial data from the clinical Phase 1 trial reported promising results,^25^ but the continued Phase 2b trial failed to improve the clinical course of patients with HF and reduced EF.^26^ It is therefore likely that other components, especially in the functional regulation of SERCA2a, contribute to the overall function of SR Ca^2+^ uptake and are therefore integral for treatment to succeed. One key mechanism for the regulation of SERCA2a is PLB that upon inhibitory binding to SERCA2a inhibits the Ca^2+^ transport of the pump; therefore, we analysed expression levels of PLB and determined the ratio between the two. This comparison did not strengthen our explanation of reduced SERCA2a function as PLB was unchanged between the groups and the ratio between PLB/SERCA2a only revealed a strong tendency (*P* = 0.055). PLB interaction with SERCA2a relies, however, on the phosphorylation status of PLB. We found a significant lower expression of phosphorylated PLB at both Ser‐16 and Thr‐17 in MI hearts. Phosphorylation of both the protein kinase A (PKA) site Ser‐16 and the CaMKII site Thr‐17 relieves the PLB inhibition of SERCA2 and thus increases its capacity to remove cytosolic Ca^2+^. Although reduced PLB phosphorylation is regarded as an important mechanism of lower SERCA2a activity in HF, conflicting results exist from both animal models and the limited work done in human HF; human data from explanted dilated cardiomyopathy hearts with terminal HF found both Ser‐16 and Thr‐17 phosphorylations of PLB to be reduced,^15^, ^16^ but a later report found only Ser‐16, but not Thr‐17, phosphorylation to be reduced.^17^

To further determine the upstream signalling of reduced PLB phosphorylation at Thr‐17, we analysed the activity of CaMKII by measuring phosphorylated CaMKII at the Thr‐286 site. pCaMKII‐Thr‐286 increases the affinity of the CaMKII complex and traps CaMKII on the autophosphorylated subunit. As a result, the kinase retains close to full activity as long as CaM is trapped, regardless of the Ca^2+^ concentration. Our data display significantly reduced pCaMKII in post‐MI HF that may further explain the dephosphorylated Thr‐17 PLB in these patients. The reduced CaMKII phosphorylation in mid‐phase stage HF patients in the present study is not easy to reconcile with earlier studies from both experimental animal models (reviewed by, e.g. Maier and Bers^27^) and explanted end‐stage human hearts showing augmented levels and activity of CaMKII.^28^, ^29^ However, more recent reports indicate controversies in terms of the activity of CaMKII in HF; data from explanted end‐stage failing human hearts show an important role of increased CaMKII activity measured by significantly increased RyR phosphorylation on the Ser‐2814 site,^30^ but importantly, this study did on the contrary not find increased levels of CaMKII phosphorylation on the RyR in severe aortic stenosis patients with compensated hypertrophy, indicating that CaMKII is not activated at earlier stages of HF in these patients.^30^ Furthermore, a different study reported that CaMKII activity was increased in explanted failing hearts with dilated cardiomyopathy but not in patients with ischaemic cardiomyopathy, whereas other labs report significantly increased CaMKII levels in explanted ischaemic cardiomyopathy hearts.^31^ Furthermore, CaMKII phosphorylation was also examined because of the previously noted role in causing RyR Ca^2+^ leaks,^30^ which could support our findings of increased spontaneous Ca^2+^ releases in MI. Given that we found reduced CaMKII Thr‐286 phosphorylation, other mechanisms for a potential RyR leak are probably present. In 2000, Marks *et al*. proposed that PKA hyperphosphorylates the RyR2 at Ser‐2808 site, causing FK506 binding protein 12.6 (FKBP 12.6) dissociation from the RyR, with subsequent diastolic SR Ca^2+^ leak. This initial observation was followed up by a series of reports supporting the hypothesis,^32^, ^33^, ^34^ though it has however been challenged in recent years.^35^, ^36^, ^37^ Our results indicate that it is unlikely that PKA can explain the observed increased in spontaneous Ca^2+^ release events, given that pPLB Ser‐16 (which is the PKA phosphosite of PLB) was almost undetectable in biopsies from MI patients. Other candidates therefore include accessory protein‐mediated effects; for example, S100A and sorcin are both Ca^2+^ binding proteins known to modulate RyR open probability independent of phosphorylation status.^38^, ^39^, ^40^ Moreover, several of the proteins involved in the excitation contraction coupling of cardiomyocytes have been shown to be influenced by oxidative modifications.^41^ Differences in antioxidant enzyme activity and oxidative stress in the MI patients, previously established,^42^ could possibly alter the activation state of CaMKII, as oxidation of CaMKII (at methionine 281/282) increases its activity and consequently causes more leaky RyR channels independent of Thr‐286 phosphorylation.^43^ We were unfortunately not able to explore these mechanisms in our limited biopsy material. Further studies are therefore warranted to fully explain the aetiology and phenotype of mid‐stage post‐MI HF in human heart. Nonetheless, these studies indicate that there are large discrepancies and controversies on protein regulation and their phosphorylation status in HF. This discrepancy may be linked to both stages of the disease and the different aetiologies of HF; both these factors should therefore be considered carefully.

### Transverse‐tubule density and distribution

The presence of a dense network of T‐tubules connected to the plasma membrane ensures a rapid and coordinated RyR Ca^2+^ release during systole,^44^ whereas loss or disorganization of T‐tubules contributes to impaired Ca^2+^ cycling and contractile failure.^45^ Disrupted T‐tubule density has previously been demonstrated in explanted end‐stage HF human hearts from different aetiologies, also including ischaemic heart disease.^2^, ^45^, ^46^, ^47^, ^48^, ^49^ Data from both larger animal models like pig and dogs^49^, ^50^ and smaller animal models like mice and rats report disrupted T‐tubule density also in earlier stages of the disease.^51^ Data from the present study confirm therefore that T‐tubule disorganization and loss are present also at earlier, not terminal, stages of human post‐MI HF. Although we do not directly assess the functional consequence of the loss, the observed slower time to Ca^2+^ transient may indicate reduced coupling between membrane excitation and SR Ca^2+^ release, because of disrupted cleft spacing between the L‐type Ca^2+^ channels and RyRs.^49^

In conclusion, for the first time, viable and intact cardiomyocytes have successfully been isolated from the mid‐myocardium in remote, non‐infarct areas of the left ventricle of human hearts at a stage of post‐MI and mid‐phase of HF, before the condition has decompensated and reached end‐stage HF. This is an obvious departure from previously published studies of HF in human patients^21^ or animal studies, of which large animal models utilizing dogs or rabbits have been thought to provide the most clinically useful replacements.^11^ Nonetheless, although large animal models present with important similarities to humans, for example, with respect to excitation contraction coupling and contractile mechanisms, they also differ with respect to aetiology and have been reported to differ in aspects of cardiac function and response to injury;^52^, ^53^, ^54^ see also the review by Hasenfuss.^11^

Functional assessments of the human left ventricle cardiomyocytes in the current study reveal contractile and intracellular Ca^2+^ handling dysfunction. Depressed cardiomyocyte function was associated with impaired systolic and diastolic intracellular and SR Ca^2+^ handling, probably due to impaired SERCA2a activity. This results from a combination of alteration in PLB/SERCA2a ratio and chronic dephosphorylation of PLB as well as loss of T‐tubules, which disrupts normal intracellular Ca^2+^ homeostasis and handling. To our knowledge, these mechanisms are not previously explored in human left ventricle myocardial samples from MI patients at mid‐stage of HF. The controversies existing on the cellular mechanisms explored in the present paper further highlight the importance of considering both the stages and the aetiology of the disease.

## Conflict of interest

None declared

## Funding

K.G. Jebsen Family Foundation; Norwegian Council of Cardiovascular Disease, the Norwegian Research Council (Young Outstanding Investigators); Funds for Cardiovascular and Medical Research at St Olav's University Hospital, Trondheim; Simon Fougner Hartmanns Family Foundation; The British Heart Foundation; Fondation Leducq Award to the Alliance for Calmodulin Kinase Signaling in Heart Disease; The Liaison Committee between the Central Norway Regional Health Authority (RHA); the Norwegian University of Science and Technology (NTNU).
